# Condition-Specific Healthcare Expenditures for Treated Knee Injuries and Shoulder Disorders in the Post-Pandemic United States

**DOI:** 10.3390/healthcare14111591

**Published:** 2026-06-05

**Authors:** Man Hung, Annabella Jensen, Isabella Strickler, Jaysen Jensen

**Affiliations:** 1 College of Dental Medicine, Roseman University of Health Sciences, South Jordan, UT 84095, USA; 2 Spencer Fox Eccles School of Medicine, University of Utah, Salt Lake City, UT 84112, USA; 3 William Beaumont School of Medicine, Oakland University, Rochester, MI 48309, USA; 4 Data Science Institute, University of Chicago, Chicago, IL 60637, USA

**Keywords:** musculoskeletal disorders, knee injury, shoulder disorders, Medical Expenditure Panel Survey, health expenditures

## Abstract

**Introduction:** Musculoskeletal conditions impose a substantial economic burden on the United States (U.S.) healthcare system, but contemporary national estimates of condition-specific spending for common orthopaedic conditions remain limited. This study utilized the 2023 Medical Expenditure Panel Survey (MEPS) to estimate the national prevalence, condition-specific expenditures, and payer distribution for treated knee injuries and shoulder disorders. **Methods:** Adults with treated knee injuries or shoulder disorders were identified using ICD-10-CM codes from the MEPS Medical Conditions File. Condition-specific expenditures were estimated by linking diagnoses to medical events and payments using the MEPS Condition–Event Link File. Expenditures were aggregated across inpatient, outpatient, office-based, emergency, home health, and prescribed medicine categories. Survey-weighted analyses were used to estimate national prevalence, mean expenditures, service-level spending patterns, and payer distributions. Survey-weighted Gamma generalized linear models with log link were used to examine patient characteristics associated with expenditures among the U.S. civilian noninstitutionalized population with positive condition-specific spending. **Results:** The analysis identified 2.55 million adults with treated knee injuries and 2.58 million adults with treated shoulder disorders. Mean annual condition-specific expenditures per person were higher for knee injuries ($10,552; 95% CI: $6128–$14,975) than for shoulder disorders ($4310; 95% CI: $3337–$5283). Knee injury expenditures were concentrated in inpatient and home health care, whereas shoulder disorder expenditures were concentrated in outpatient and office-based care. Private insurance, Medicare, out-of-pocket payments, and Worker’s Compensation each contributed to the financial burden, with payer distributions varying by condition. In adjusted models, fair/poor self-rated health and female sex were associated with higher knee injury expenditures, while no covariates were statistically significant for shoulder disorder expenditures. **Conclusions:** Treated knee injuries and shoulder disorders showed distinct condition-specific expenditure profiles across care settings and payer sources. These findings provide contemporary national benchmarks for orthopaedic spending and may support future research, utilization monitoring, and value-based reimbursement planning.

## 1. Introduction

Musculoskeletal disorders represent one of the most significant public health and economic burdens facing the United States (U.S.) healthcare system today [1,2]. As a leading cause of disability and a primary driver of medical care utilization, orthopaedic conditions account for hundreds of billions of dollars in annual healthcare expenditures, rivaling the economic impact of cardiovascular disease and diabetes [2,3]. Throughout this study, the term ‘expenditures’ refers to payments made by insurers, public programs, or patients for healthcare services rather than provider-incurred production costs. Within this broad category, injuries and degenerative disorders of the large joints, specifically the knee and shoulder, comprise a disproportionate share of clinical volume and resource use [4,5,6]. The rising prevalence of these conditions is propelled by converging demographic trends, including an aging population, increasing rates of obesity, and sustained participation in sports and occupational activities among younger adults [4,6,7]. Consequently, understanding the granular economic profile of these conditions is essential for policymakers, payers, and clinicians seeking to optimize resource allocation and advance value-based care delivery [2,6,7].

Knee and shoulder pathologies manifest across a wide spectrum of severity, ranging from acute ligamentous and meniscal injuries to chronic, degenerative osteoarthritis requiring surgical intervention. The management of these conditions has evolved substantially over the past decade, characterized by increasing reliance on ambulatory surgery centers and office-based care for many shoulder procedures, alongside the continued need for resource-intensive inpatient services for complex knee reconstruction, arthroplasty, and trauma [8,9,10]. While these shifts have implications for cost containment and efficiency, their true economic impact remains difficult to quantify. Knee and shoulder conditions were selected because they represent two high-volume orthopaedic categories with distinct treatment pathways, care settings, and payer distributions, allowing comparison of differing expenditure structures within musculoskeletal care. Administrative claims databases provide detailed procedural cost information but often lack comprehensive capture of patient-reported out-of-pocket (OOP) spending, indirect cost burden, and socioeconomic context [11,12]. Moreover, aggregate expenditure analyses frequently fail to distinguish between all-cause healthcare spending and costs directly attributable to a specific orthopaedic diagnosis, resulting in imprecise estimates of disease-specific economic burden [1,12,13].

Musculoskeletal conditions impose a substantial economic burden on the U.S. healthcare system. Prior analyses using the Medical Expenditure Panel Survey (MEPS) have demonstrated that individuals with arthritis and joint pain incur significantly higher annual healthcare expenditures compared with those without musculoskeletal conditions, even after adjustment for sociodemographic factors and functional limitations [1,2,13]. These findings established musculoskeletal disease as a major driver of national healthcare spending and supported the use of MEPS as a useful platform for condition-specific expenditure analyses. However, much of the existing literature predates the COVID-19 pandemic and lacks contemporary estimates for discrete anatomic conditions such as knee injuries and shoulder disorders, which may limit applicability to current healthcare delivery and payment environments.

The payer landscape further complicates economic evaluation of orthopaedic care. Although Medicare bears a substantial share of costs related to age-associated joint degeneration, private insurance and Worker’s Compensation programs play a critical and often under-examined role in financing care for working-age individuals with knee and shoulder disorders [5,14]. This distinction is particularly salient for shoulder pathology, which is more commonly managed in privately insured populations and outpatient settings, whereas knee injuries are more frequently associated with greater comorbidity burden and higher healthcare utilization. As healthcare systems increasingly transition toward bundled payments, episode-based reimbursement, and risk-sharing models, there is a growing need for accurate, contemporary, and nationally representative benchmarks of condition-specific spending that reflect real-world patterns of care across payer types [15].

To address these gaps, this study leveraged data from the 2023 MEPS to provide an updated and comprehensive economic analysis of treated knee injuries and shoulder disorders in the U.S. civilian noninstitutionalized population. Although reliance on a single calendar year may raise concerns regarding temporal variability, the use of 2023 MEPS data offers several important methodological and policy advantages. MEPS is uniquely designed to generate nationally representative annual estimates of healthcare utilization, expenditures, insurance coverage, and patient OOP costs by integrating data from household surveys, medical providers, and pharmacies [6,11,12]. This structure enables precise attribution of expenditures to specific diagnostic conditions while preserving detailed sociodemographic and payer information not available in claims-based datasets.

Importantly, the 2023 MEPS represents one of the earliest data years to reflect an early post-pandemic healthcare environment, following the widespread disruptions [16,17] in elective care, insurance coverage, and healthcare utilization observed during earlier phases of the COVID-19 pandemic [1]. Orthopaedic care was directly affected by these disruptions, as many nonurgent evaluations and procedures were delayed, rescheduled, or shifted across outpatient, telehealth, and other care settings. As such, expenditure estimates derived from 2023 data provide an updated national benchmark of knee and shoulder condition-specific spending during a period when healthcare delivery patterns were re-stabilizing after pandemic-related disruption [18]. Furthermore, single-year MEPS analyses are widely accepted in health services and health economics research when the objective is to establish current national benchmarks, evaluate payer-specific spending, or inform near-term policy decisions, particularly in periods of rapid system change [12,14,19].

Accordingly, the objectives of this study were to: (1) quantify annual condition-specific expenditures associated with treated knee injuries and shoulder disorders, stratified by service setting; (2) examine the distribution of financial burden across major payers, including the often-under-recognized contribution of Worker’s Compensation; and (3) examine sociodemographic and clinical factors associated with conditions-specific expenditures using multivariable Gamma generalized linear model (GLM). By leveraging the most recent nationally representative data and isolating expenditures directly linked to orthopaedic diagnoses, this study provides contemporary national estimates of the economic burden of knee and shoulder conditions.

## 2. Methods

### 2.1. Data Source

This retrospective cross-sectional study utilized data from the 2023 MEPS Household Component (HC), a nationally representative survey sponsored by the Agency for Healthcare Research and Quality [11,12]. The MEPS-HC collects comprehensive data on health care utilization, expenditures, payment sources, and health status for the U.S. civilian noninstitutionalized population, using an overlapping panel design drawn from the National Health Interview Survey. The analytical dataset was constructed by linking the Medical Conditions File (HC-249), which provides diagnostic information; the Full Year Consolidated Data File (HC-251), which contains patient-level demographic, socioeconomic, insurance, and survey design variables; and MEPS Event Files, which capture utilization and expenditures across inpatient hospital stays (HC-248D), outpatient department visits (HC-248F), office-based medical provider visits (HC-248G), emergency room visits (HC-248E), and home health visits (HC-248H), and prescribed medicines (HC-248A). The Condition–Event Link File (HC-248IF1) was employed to link specific medical events and their associated expenditures to the identified orthopaedic conditions, ensuring that expenditures were attributed to the condition of interest rather than to all-cause healthcare use.

### 2.2. Study Population and Cohort Identification

The study population comprised all adults aged 18 years and older with at least one medical record associated with the target orthopaedic conditions during the 2023 calendar year. Diagnostic cohorts were identified using ICD-10-CM codes reported in the Medical Conditions File. The knee injury cohort was defined by codes S82 (fracture of lower leg, including ankle), S83 (dislocation and sprain of joints and ligaments of knee), and M23 (internal derangement of knee). The shoulder disorders cohort was defined by codes M75 (shoulder lesions), S42 (Fracture of shoulder and upper arm), S43 (Dislocation and sprain of joints and ligaments of shoulder girdle), and S46 (injury of muscle, fascia, and tendon at shoulder and upper arm level).

### 2.3. Outcome Measures

The primary outcome measure was annual condition-specific medical expenditures, defined as the sum of direct payments from all sources for medical events explicitly linked to the index condition. Payment sources included OOP, private insurance, Medicare, Medicaid, Worker’s Compensation, and other federal or state sources (e.g., VA, TRICARE). Expenditures were aggregated across inpatient hospital, outpatient facility, office-based, emergency room, home health, and prescribed medicine categories. Each service category was reported separately, and total expenditures were calculated as the sum of all linked condition-specific payments across these categories. This approach distinguishes the direct economic burden of treating the specific orthopaedic injury from the patient’s total annual healthcare spending, providing a more precise estimate of disease burden.

### 2.4. Covariates

Patient-level characteristics were extracted from the HC-251 to serve as covariates in the multivariable analysis. Age was treated as a continuous variable, and sex was dichotomized into male or female. Race and ethnicity were categorized into Non-Hispanic White, Non-Hispanic Black, Hispanic, and Other/Multiple Races based on the RACETHX variable. Insurance status was derived from the INSURC23 variable and hierarchically assigned as Any Private, Public Only (Medicare, Medicaid, TRICARE, or VA), or Uninsured. Socioeconomic Status (SES) was assessed using the POVCAT23 variable, with “poor/low income” defined as a family income less than 200% of the federal poverty level. Health status was assessed using the perceived health status variable (RTHLTH53) and dichotomized into “fair/poor” versus “good/very good/excellent” to serve as a proxy for comorbidity burden.

### 2.5. Statistical Analyses

All statistical analyses accounted for the complex survey design of MEPS, including stratification, clustering, and weighting, to generate nationally representative estimates [16]. Descriptive statistics, including weighted means and proportions, were calculated for demographic characteristics and mean annual expenditures per person, stratified by condition and service type. For weighted expenditure estimates, standard errors (SEs), 95% confidence intervals (CIs), and relative standard errors (RSEs) were calculated using the MEPS final person weight (PERWT23F), variance strata (VARSTR), and primary sampling units (VARPSU). RSEs were used to evaluate estimate precision, with values greater than 30% indicating reduced reliability. Between-cohort differences in mean expenditures were assessed using design-based SEs, 95% CIs, and *p*-values. To evaluate the robustness of the knee injury cohort definition, we conducted a sensitivity analysis excluding S82-coded conditions, because S82 may include lower-leg fractures extending beyond isolated knee pathology. The primary knee-versus-shoulder expenditure comparison was repeated using this S82-excluded knee cohort.

To examine patient characteristics associated with condition-specific expenditures, we developed a GLM with a Gamma distribution and a log link function. This model specification is standard in health economics for analyzing skewed cost data with a long right tail [7,16]. The model was applied to the subset of patients with positive condition-specific expenditures. Thus, the model estimated adjusted associations with expenditure levels among individuals who had positive condition-specific spending, rather than the probability of incurring any expenditure. To preserve model parsimony, independent variables were limited to age, sex, race, insurance, SES, and health status. Coefficients from the model represent the change in the log of expenditures, and exponentiated coefficients can be interpreted as cost multipliers. All data processing and statistical analyses were performed using Python (v3.10) with the pandas and statsmodels libraries, utilizing the PERWT23F for all population estimates.

## 3. Results

### 3.1. Study Population Characteristics

The analysis of the 2023 MEPS identified two primary orthopaedic cohorts representing a substantial segment of the U.S. civilian noninstitutionalized population (Table 1). The study identified 155 unweighted cases of treated knee injuries, representing a weighted national population of approximately 2.55 million adults, and 165 unweighted cases of shoulder disorders, representing 2.58 million adults. Although unweighted sample sizes were modest, application of MEPS weights enabled generation of nationally representative population estimates. Demographic and clinical profiles for the available cohorts are detailed in Table 2. Patients treated for knee injuries were, on average, older than those treated for shoulder disorders (mean age 53.9 years vs. 50.2 years). Both cohorts were predominantly female (57.4% for knee injuries; 55.0% for shoulder disorders) and Non-Hispanic White (72.2% and 81.2%, respectively). Socioeconomic disparities were notable, with 25.6% of the shoulder disorders cohort classified as poor or low income (below 200% of the Federal Poverty Level), compared to 17.3% of the knee injury cohort. A significant burden of comorbidity was observed across both groups, with 16.0% of knee patients and 18.2% of shoulder patients reporting fair or poor self-rated health. Insurance coverage patterns were broadly similar, with approximately half of each cohort holding private insurance and roughly 15% relying solely on public coverage (Medicare or Medicaid).

### 3.2. Condition-Specific Medical Expenditures

**Figure 1:**
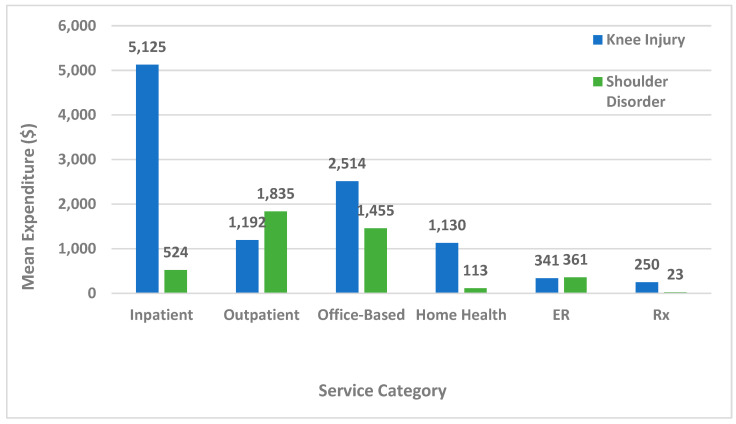
Mean annual condition-specific expenditures per person by service category.

Estimates of annual direct medical expenditures attributable to the treated conditions are presented in Table 3 and Figure 1. Mean per-person condition-specific expenditure was higher for Knee Injuries ($10,552; SE = $2183; 95% CI: $6128–$14,975; RSE = 20.7%) than for Shoulder Disorders ($4310; SE = $477; 95% CI: $3337–$5283; RSE = 11.1%). The weighted mean difference was $6242 (SE = $2325; 95% CI: $1606–$10,877; *p* = 0.009; RSE = 37.2%). Across service categories, knee injury expenditures were greatest for inpatient care, while shoulder disorder expenditures were concentrated in outpatient and office-based care. Home health expenditures were also higher for knee injuries than for shoulder disorders. Several service-specific estimates had RSEs greater than 30%, including knee inpatient and home health expenditures and shoulder inpatient, emergency room, home health, and prescribed medicine expenditures, indicating reduced precision for these estimates.

In the S82 sensitivity analysis, excluding S82-coded conditions reduced the knee injury cohort from 155 to 81 unweighted persons. The weighted mean total knee expenditure decreased from $10,552 to $5234 (SE = $1191; 95% CI: $2882–$7586; RSE = 22.8%). After excluding S82, the knee-versus-shoulder expenditure difference was no longer statistically significant (difference = $924; SE = $1160; 95% CI: −$1402–$3251; *p* = 0.429; RSE = 125.5%).

### 3.3. Payer Distribution

**Figure 2:**
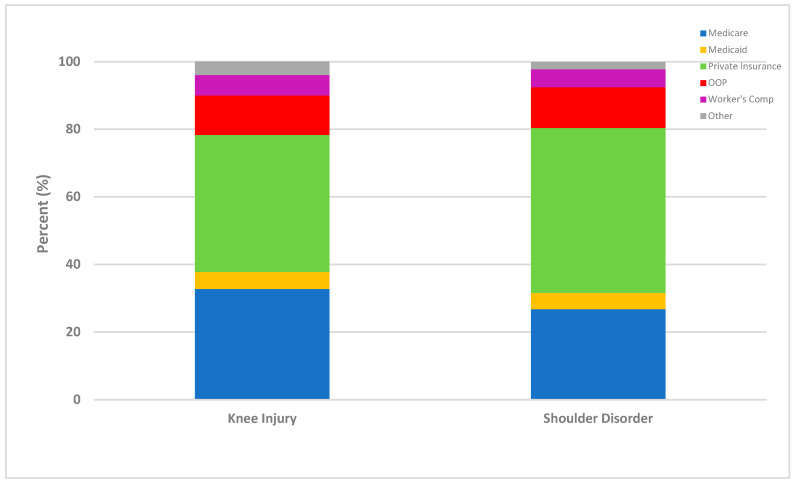
Payer distribution of annual condition-specific expenditures.

The payer distribution, presented in Table 4 and Figure 2, highlighted the financial burden shared across different sources. Private insurance remained the primary payer for both conditions, covering 40.5% of costs for knee injuries and 48.8% for shoulder disorders. Medicare covered a larger share of expenditures for knee injuries (32.8%) than for shoulder disorders (26.8%). OOP spending was similar across both groups, accounting for approximately 11.7% to 12.0% of total expenditures. Worker’s Compensation contributed to condition-specific expenditures, covering 6.0% of costs for knee injuries and 5.3% for shoulder disorders. These findings indicate that the financial burden of treated knee and shoulder conditions is distributed across public programs, private insurance, patients, and occupational payers.

### 3.4. Predictors of High Expenditures

A survey-weighted Gamma GLM with a log link function was used to examine patient characteristics associated with expenditures among individuals with positive condition-specific spending (Table 5). For knee injuries, fair/poor self-rated health was significantly associated with higher expenditures (coefficient = 1.205; SE = 0.333; 95% CI: 0.553–1.857; *p* = 0.0003), and female sex was also significant (coefficient = 0.731; SE = 0.365; 95% CI: 0.015–1.447; *p* = 0.045). For shoulder disorders, no covariates reached statistical significance in the adjusted model. Table 5 presents the full model results.

## 4. Discussion

### 4.1. The Economic Divergence of Orthopaedic Trauma and Degeneration

This study provides a contemporary national estimate of condition-specific expenditures for treated knee injuries and shoulder disorders using 2023 MEPS data. Prior MEPS-based and national expenditure studies have shown that arthritis, joint pain, chronic pain, and other musculoskeletal conditions are associated with substantial healthcare spending and economic burden [1,2,3,5,13]. More recent work has also emphasized the continued population-level burden of musculoskeletal disease and the need for updated health services and economic estimates [4,6,7]. Building on this literature, the present study focuses on two common orthopaedic condition groups.

The two groups showed distinct expenditure profiles across service settings. Knee injury expenditures were higher in the primary analysis and were concentrated in inpatient and post-acute care, whereas shoulder disorder expenditures were concentrated in outpatient and office-based care. These patterns are consistent with differences in care pathways for traumatic or surgically managed lower-extremity injuries versus many shoulder conditions managed in ambulatory settings. At the same time, the S82 sensitivity analysis showed that the broad knee injury definition materially influenced the magnitude of the knee-versus-shoulder expenditure difference, underscoring the importance of interpreting the knee cohort as a broad treated-knee/lower-extremity-injury group rather than as isolated knee pathology alone.

### 4.2. The “Health Penalty” in Knee Care

In the adjusted exploratory models, fair/poor self-rated health was associated with higher knee injury expenditures. This finding is consistent with the broader pattern of higher inpatient and home health spending observed in the knee cohort and may reflect greater clinical complexity among individuals with poorer overall health. Female sex was also associated with higher knee injury expenditures in the adjusted model. Because of the modest unweighted cohort size and the model’s restriction to individuals with positive condition-specific spending, these associations should be interpreted as exploratory rather than causal.

### 4.3. Supply-Sensitive Demand in Shoulder Management

Shoulder disorder expenditures showed a more ambulatory profile, with spending concentrated in outpatient and office-based settings. No covariates reached statistical significance in the adjusted shoulder disorder model, suggesting that the available MEPS sample may have limited power to detect predictors of shoulder-specific expenditures after adjustment. Accordingly, the shoulder findings are best interpreted as descriptive national estimates of service-specific spending rather than definitive evidence of individual-level cost predictors.

### 4.4. The Hidden Role of Occupational Health

The payer analysis highlights the role of occupational health within condition-specific orthopaedic spending. Worker’s Compensation accounted for 6.0% of knee injury expenditures and 5.3% of shoulder disorder expenditures, indicating that occupational payers contribute meaningfully to the financing of treated musculoskeletal conditions. Although MEPS captures direct medical payments, it does not estimate related indirect costs such as lost productivity, wage replacement, disability payments, or delayed return to work. These unmeasured costs may be important for understanding the broader societal burden of orthopaedic injuries.

### 4.5. Strengths and Limitations

This study has several notable strengths. First, the use of the 2023 MEPS provides the most current nationally representative estimates of orthopaedic expenditures available, offering a post-pandemic snapshot of healthcare utilization. Second, unlike studies that rely solely on administrative claims from a single payer (e.g., Medicare), this study captures the full spectrum of payment sources, including private insurance, Medicare, Medicaid, OOP spending, and the often-fragmented Worker’s Compensation system. Third, the methodology leverages the MEPS Condition–Event Link File to attribute medical expenditures directly to the orthopaedic condition of interest, enabling isolation of condition-specific costs rather than all-cause healthcare spending. This approach reduces inflation from unrelated comorbid care (e.g., diabetes or cardiovascular disease management) and provides a more precise estimate of the economic burden attributable to treated knee and shoulder conditions. Unlike single-payer claims databases, MEPS captures OOP and Worker’s Compensation payments, enabling a more complete estimate of the national economic burden of orthopaedic conditions [11,12].

These findings should be interpreted in light of several limitations. First, this analysis grouped knee injuries and shoulder disorders across a heterogeneous spectrum of traumatic and degenerative diagnoses, which may introduce clinical variability within each cohort. This issue is particularly relevant for the knee cohort because the S82 category includes lower-leg fractures that may extend beyond isolated knee pathology. In sensitivity analysis, excluding S82-coded conditions reduced the estimated mean knee expenditure and attenuated the knee-versus-shoulder expenditure difference, indicating that the primary knee estimates reflect a broader treated-knee/lower-extremity-injury group rather than a narrowly defined knee-only cohort. However, this approach reflects real-world treatment and reimbursement patterns, where orthopaedic care is frequently organized and financed at the anatomic or episode level rather than by isolated diagnostic entities. Importantly, the use of the MEPS Condition–Event Link File allows expenditures to be attributed specifically to the indexed condition, mitigating bias from unrelated healthcare utilization and supporting valid estimation of condition-specific economic burden at the national level.

Second, as a cross-sectional survey, MEPS captures the healthcare expenditures incurred within a single calendar year and may not fully reflect the complete longitudinal episode of care for injuries occurring late in the year. Additionally, longer-term downstream costs, such as revision surgery, prolonged rehabilitation, or chronic pain management extending beyond the survey year, are not captured. Although the unweighted sample sizes for each cohort were modest, which may limit statistical power and coefficient stability in multivariable analyses, all analyses incorporated MEPS weights and design variables to generate nationally representative estimates. The modest cohort sizes are especially relevant for the multivariable Gamma regression models, which may be sensitive to sparse covariate patterns. In addition, because the regression analyses were restricted to individuals with positive condition-specific expenditures, the models describe expenditure levels among treated individuals with spending rather than the probability of incurring any expenditure. Accordingly, the findings should be interpreted as population-level estimates of condition-specific expenditures rather than precise estimates of rare or infrequent events. The descriptive expenditure estimates and sensitivity analyses provide the primary evidence from this study, while regression-based findings should be interpreted as exploratory associations. Despite these limitations, MEPS remains a robust and widely accepted platform for generating contemporary national benchmarks of healthcare utilization and spending.

### 4.6. Implications for Policy and Practice

The observed differences in expenditure patterns between knee injuries and shoulder disorders suggest that policy and practice implications should be condition-specific rather than based on a single musculoskeletal cost-containment strategy. The primary implication is not simply to reduce spending indiscriminately or renegotiate insurance premiums, but to identify where potentially avoidable or modifiable expenditures may occur and where payment models should better reflect clinical complexity. For knee injuries, expenditures were concentrated in inpatient and post-acute care settings, suggesting that bundled payment models, episode-based reimbursement, and quality metrics should incorporate adequate risk adjustment for patient frailty, comorbidity burden, and injury severity. Without such adjustment, payment models that reward lower spending may inadvertently penalize providers caring for medically complex patients who legitimately require higher-intensity inpatient or home health services.

For shoulder disorders, expenditures were concentrated in outpatient and office-based settings and were more closely associated with private insurance coverage. These findings suggest that policy efforts may be better directed toward appropriate use criteria for diagnostic imaging, physical therapy, injections, and elective procedures; price transparency for outpatient services; and evaluation of site-of-care variation in commercially insured populations. In this context, the findings may inform payer negotiations and benefit design indirectly, but they should not be interpreted as evidence that insurance premiums should be renegotiated solely on the basis of these estimates. Rather, the results provide national benchmarks that payers, employers, and health systems can use to evaluate whether spending patterns are consistent with expected care pathways and value-based care goals.

The contribution of Worker’s Compensation also has practical implications. The share of expenditures paid by Worker’s Compensation suggests that treated knee and shoulder conditions may impose a meaningful occupational health burden. Employers, occupational health programs, and Worker’s Compensation payers may use these findings to prioritize injury prevention, ergonomic interventions, early rehabilitation, and return-to-work programs. Such interventions may reduce avoidable medical expenditures and indirect productivity losses, although the present MEPS analysis captures only direct medical payments and does not estimate wage replacement, absenteeism, presenteeism, or long-term disability costs.

### 4.7. Future Directions

Future research should prioritize longitudinal analyses that track the full “episode of care” across multiple years to capture the long-term economic trajectory of orthopaedic injuries, including the costs of revision surgery and chronic disability. Linking MEPS data with detailed administrative claims could also help overcome sample size limitations and support more detailed analyses of specific diagnoses, procedures, and care pathways. Future economic evaluations should also incorporate indirect costs, including absenteeism and presenteeism, wage replacement, and disability payments, to provide a more complete understanding of how musculoskeletal health affect patients, employers, and the broader U.S. economy.

## 5. Conclusions

This study provides contemporary national estimates of condition-specific healthcare expenditures for treated knee injuries and shoulder disorders. The analysis showed that knee injuries were associated with greater inpatient and post-acute care spending, whereas shoulder disorders were associated with a more ambulatory expenditure profile concentrated in outpatient and office-based care. These differences suggest that musculoskeletal expenditure patterns vary meaningfully by condition type and care setting. By linking medical conditions to service-specific events and payments, this study provides updated benchmarks that may support future research, utilization monitoring, and value-based reimbursement planning for orthopaedic care.

## Figures and Tables

**Table 1:** Orthopaedic condition cohort definitions.

Condition Cohort	ICD-10-CM Codes Identified	Unweighted N *	Weighted N **
Knee Injury	S82 (Fracture of lower leg) S83 (Dislocation/sprain of knee) M23 (Internal derangement of knee)	155	2,546,618
Shoulder Disorders	M75 (Shoulder lesions) S42 (Fracture of shoulder/upper arm) S43 (Dislocation/sprain of shoulder) S46 (Injury to muscle/tendon of shoulder)	165	2,584,051

Note: * Survey sample size. ** Weighted estimates representing the U.S. civilian noninstitutionalized population. Cohort definitions were intended to capture broad clinically managed orthopaedic conditions and may include heterogeneous traumatic and degenerative diagnoses.

**Table 2:** Demographic and clinical characteristics of U.S. adults with orthopaedic conditions.

Characteristic	Knee Injury	Shoulder Disorder
Mean Age	53.9 Years	50.2 Years
Sex		
Female	57.4%	55.0%
Race/Ethnicity		
Non-Hispanic White	72.2%	81.2%
Non-Hispanic Black	9.6%	6.2%
Hispanic	8.8%	8.3%
Insurance Status		
Any Private	54.1%	48.8%
Public Only (Medicare/Medicaid)	14.6%	14.5%
Uninsured	1.6%	0.6%
Socioeconomic Status		
Poor/Low Income (<200% federal poverty level)	17.3%	25.6%
Health Status		
Fair/Poor Self-Rated Health	16.0%	18.2%

**Table 3:** Mean annual condition-specific expenditures per person (USD).

Service Category	Knee Injury	Shoulder Disorder
Total Expenditures	$10,552 (SE = $2183; 95% CI: $6128–$14,975; RSE = 20.7%)	$4310 (SE = $477; 95% CI: $3337–$5283; RSE = 11.1%)
Inpatient (Hospital)	$5125 (48.6%; SE = $1543; 95% CI: $1999–$8251; RSE = 30.1%)	$524 (12.2%; SE = $172; 95% CI: $173–$875; RSE = 32.8%)
Outpatient (Facility)	$1192 (11.3%; SE = $299; 95% CI: $586–$1798; RSE = 25.1%)	$1835 (42.6%; SE = $321; 95% CI: $1179–$2490; RSE = 17.5%)
Office-Based Visits	$2514 (23.8%; SE = $508; 95% CI: $1485–$3543; RSE = 20.2%)	$1455 (33.8%; SE = $254; 95% CI: $937–$1972; RSE = 17.4%)
Prescribed Medicines	$250 (2.4%; SE = $159; 95% CI: −$72–$572; RSE = 63.5%)	$23 (0.5%; SE = $11; 95% CI: −$0–$46; RSE = 50.1%)
Home Health	$1130 (10.7%; SE = $389; 95% CI: $342–$1918; RSE = 34.4%)	$113 (2.6%; SE = $67; 95% CI: −$25–$250; RSE = 59.8%)
Emergency Room	$341 (3.2%; SE = $79; 95% CI: $181–$501; RSE = 23.2%)	$361 (8.4%; SE = $123; 95% CI: $111–$612; RSE = 34.0%)

**Table 4:** Distribution of payer source by injury cohort.

Payer Source	Knee Injury	Shoulder Disorder
Private Insurance	40.5%	48.8%
Medicare	32.8%	26.8%
Out-of-Pocket	11.7%	12.0%
Worker’s Compensation	6.0%	5.3%
Medicaid	5.0%	4.8%
Other (VA, Tricare, etc.)	4.0%	2.3%
Total	100.0%	100.0%

**Table 5:** Associations between patient characteristics and condition-specific expenditures using survey-weighted Gamma regression models. Coefficients represent estimated changes in log expenditures; cost ratios are exponentiated coefficients.

Variable	Knee Injury Coefficient (SE)	Knee 95% CI	Knee *p*-Value	Knee Cost Ratio (95% CI)	Shoulder Disorder Coefficient (SE)	Shoulder 95% CI	Shoulder *p*-Value	Shoulder Cost Ratio (95% CI)
Age	−0.010 (0.007)	−0.025 to 0.004	0.146	0.99 (0.98–1.00)	0.008 (0.009)	−0.009 to 0.025	0.361	1.01 (0.99–1.03)
Female	0.731 (0.365)	0.015 to 1.447	0.045	2.08 (1.02–4.25)	−0.275 (0.335)	−0.930 to 0.381	0.412	0.76 (0.39–1.46)
White Race	0.294 (0.326)	−0.345 to 0.933	0.367	1.34 (0.71–2.54)	0.009 (0.343)	−0.664 to 0.682	0.979	1.01 (0.51–1.98)
Private Insurance	0.448 (0.373)	−0.283 to 1.179	0.230	1.56 (0.75–3.25)	0.154 (0.376)	−0.583 to 0.890	0.682	1.17 (0.56–2.44)
Poor/Low Income	−0.275 (0.378)	−1.016 to 0.466	0.467	0.76 (0.36–1.59)	−0.388 (0.317)	−1.009 to 0.233	0.221	0.68 (0.36–1.26)
Fair/Poor Health	1.205 (0.333)	0.553 to 1.857	0.0003	3.34 (1.74–6.41)	−0.256 (0.324)	−0.891 to 0.378	0.428	0.77 (0.41–1.46)
Intercept	8.561 (0.541)	7.500 to 9.622	<0.001	—	8.116 (0.667)	6.808 to 9.423	<0.001	—

## Data Availability

The data are publicly available from the Agency for Healthcare Research and Quality Medical Expenditure Panel Survey website (https://meps.ahrq.gov/mepsweb/data_stats/download_data_files.jsp, accessed on 12 October 2025). All data files used can be accessed without restriction.
